# TRPV1 and TRPA1 in cutaneous neurogenic and chronic inflammation: pro-inflammatory response induced by their activation and their sensitization

**DOI:** 10.1007/s13238-017-0395-5

**Published:** 2017-03-31

**Authors:** Olivier Gouin, Killian L’Herondelle, Nicolas Lebonvallet, Christelle Le Gall-Ianotto, Mehdi Sakka, Virginie Buhé, Emmanuelle Plée-Gautier, Jean-Luc Carré, Luc Lefeuvre, Laurent Misery, Raphaele Le Garrec

**Affiliations:** 1 Laboratory on Interaction Neurons-Keratinocytes (LINK), University of Western Brittany, 29200 Brest, France; 2 Uriage Dermatological Laboratories, 92400 Courbevoie, France

**Keywords:** sensory nerve, neurogenic skin inflammation, inflammatory gene regulation, pruritus

## Abstract

Cutaneous neurogenic inflammation (CNI) is inflammation that is induced (or enhanced) in the skin by the release of neuropeptides from sensory nerve endings. Clinical manifestations are mainly sensory and vascular disorders such as pruritus and erythema. Transient receptor potential vanilloid 1 and ankyrin 1 (TRPV1 and TRPA1, respectively) are non-selective cation channels known to specifically participate in pain and CNI. Both TRPV1 and TRPA1 are co-expressed in a large subset of sensory nerves, where they integrate numerous noxious stimuli. It is now clear that the expression of both channels also extends far beyond the sensory nerves in the skin, occuring also in keratinocytes, mast cells, dendritic cells, and endothelial cells. In these non-neuronal cells, TRPV1 and TRPA1 also act as nociceptive sensors and potentiate the inflammatory process. This review discusses the role of TRPV1 and TRPA1 in the modulation of inflammatory genes that leads to or maintains CNI in sensory neurons and non-neuronal skin cells. In addition, this review provides a summary of current research on the intracellular sensitization pathways of both TRP channels by other endogenous inflammatory mediators that promote the self-maintenance of CNI.

## INTRODUCTION

### CNI definition, induction, and self-maintenance

Cutaneous neurogenic inflammation (CNI) is the inflammation induced (or enhanced) by an excessive release of neuropeptides such as calcitonin gene-related peptide (CGRP) and tachykinins (mainly substance P, SP) in the skin from locally or antidromically activated sensory nerve endings (Herbert and Holzer, 2002; Roosterman et al., 2006; Gouin et al., 2015).

CNI can be induced through the direct activation of receptors on sensory nerve endings by mechanical skin injuries and other exogenous stimuli, such as exposure of the skin to injurious heat or cold, ultraviolet (physical factors), chemical irritants or allergens. Endogenous stimuli, including osmotic or pH changes in the skin, can also initiate CNI (Herbert and Holzer, 2002; Roosterman et al., 2006). The released neuropeptides act on skin cells that express cognate neuropeptide receptors, including microvascular cells and resident mast cells, leading to degranulation, vasodilation, and extravasation of plasma proteins and leukocytes. Some clinical manifestations of acute CNI are localized pruritus, redness, heat, and edema (Herbert and Holzer 2002; Roosterman et al., 2006; Teresiak-Mikołajczak et al., 2013).

Moreover, neuropeptides and mast cell-released mediators can act on other neighboring target cells, including keratinocytes, dendritic cells, neutrophils and fibroblasts, leading to the disruption of skin homeostasis, with abnormal skin growth, differentiation, and/or immunomodulation (Shim et al., 2007; Trevisani et al., 2007; Wilson et al., 2013b; Patricio et al., 2015).

Furthermore, a variety of neurotrophic (e.g. nerve growth factor, NGF) or inflammatory mediators (e.g. proteases, histamine, cytokines, prostanoids) or endocannabinoids (e.g. anandamide) released by skin cells/nerve fibers or chemoattracted cells are able to further activate or sensitize these sensory receptors (Briot et al., 2009; Vellani et al., 2010; Roosterman et al., 2006; Riol-Blanco et al., 2014; Wilson et al., 2013b; Wei et al., 2012). Such positive feedback loops contribute to the enhancement of the inflammatory process and thus to self-maintained CNI (Gouin et al., 2015).

Therefore, CNI appears to be a multi-cellular network with multiple, multi-directional interactions leading to a vicious circle of processes that results in chronic inflammation (Gouin et al., 2015). Indeed, CNI is frequently involved in chronic inflammatory skin disorders, including psoriasis, atopic dermatitis (AD) (Kubanov et al., 2015; Smolyannikova et al., 2015), sensitive skin (Costa et al., 2014), rosacea (Kürkçüoğlu and Alaybeyi 1991; Salem et al., 2013), and hypertrophic scars (Akaishi et al., 2008; Kwak et al., 2014).

### Role of PARs and TRPs

Although the exocytosis of neuropeptides from large dense-core vesicles differs from that of classical neurotransmitters from small clear vesicles, it is also triggered by a rise in the cytosolic Ca^2+^ concentration (Huang and Neher 1996; Zupanc 1996; Jans et al., 2004). Two primary pathways lead to increased Ca^2+^ concentration in the cytosol, the Ca^2+^ influx associated with the opening of plasmalemmal Ca^2+^ channels and the Ca^2+^ released from intracellular stores (the endoplasmic reticulum and mitochondria). In addition to voltage-gated Ca^2+^ channels, cutaneous sensory nerves express cationic channels and G protein-coupled receptors (GPCRs), the activation of which can lead directly or indirectly to an increase in cytosolic Ca^2+^. In addition to the induction of neuropeptide release, an increase in cytosolic Ca^2+^ can also drive the regulation of the expression of several inflammatory genes, such as those encoding neuropeptides, cytokines, growth factors, prostaglandins (PG), and matrix metalloproteinases (MMPs), which have a possible role in chronic cutaneous inflammation.

**Figure 1:**
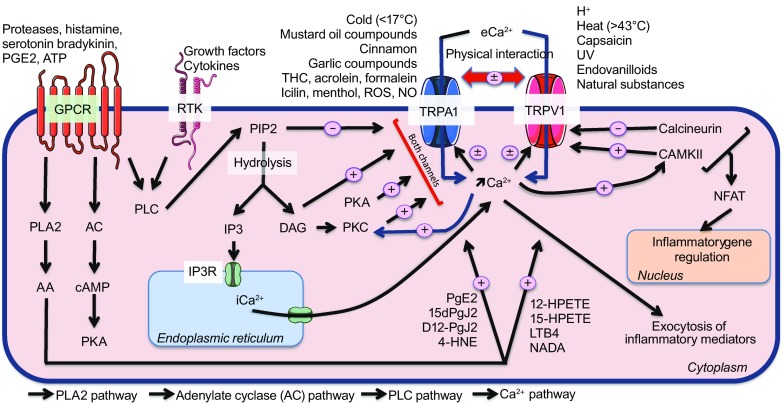
**Common intracellular GPCR, RTK and Ca** ^**2+**^ **pathways regulate via the activation and sensitization of TRPV1 and TRPA1**. The G protein-coupled receptor (GPCR) and receptor tyrosine kinase (RTK) activation stimulate phospholipase C (PLC), which leads to phosphatidylinositol 4,5-bisphosphate (PIP2) hydrolysis and the consequent release of the transient receptor potential (TRP) channels from its inhibitory control, triggering the formation of 1,4,5-trisphosphate (IP3) and diaglycerol (DAG) as well as the influx of Ca^2+^ *via* TRP vanilloid 1 and ankyrin 1 (TRPV1 and TRPA1). Then, DAG can directly activate both TRP channels, and protein kinase C (PKC) activation by DAG enhances TRP activity, sensitizing both channels. IP3 formation promotes Ca^2+^ release from the endoplasmic reticulum and increases the iCa^2+^ concentration. GPCRs can also activate the phospholipase A2 (PLA2) and adenylate cyclase pathways, which lead to protein kinase A (PKA) stimulation and the formation of arachidonic acid (AA) metabolites and products. PKA acts directly sensitize the TRP channels, while AA metabolites and products directly activate the TRP channels. Both TRPV1 and TRPV1 can directly or indirectly regulate the activity of the other by direct interaction or via the iCa^2+^ concentration, resulting in a cross-sensitization/desensitization process. The elevation of iCa^2+^ triggers the exocytosis of inflammatory mediators and stimulates both Ca^2+^/calmodulin-dependent kinase II (CAMKII) and calcineurin, which sensitize and desensitize TRPV1, respectively. Both kinases are also involved in the regulation of inflammatory genes via nuclear factor of activated T-cell (NFAT) translocation to the nucleus

Interestingly, cationic channels expressed by cutaneous nerve endings include some transient receptor potential (TRP) channels known to be involved in neuropeptide exocytosis and skin disorders that have neurogenic mechanisms. In this review we focus on two of these channels including TRPV1 and TRPA1 (TRP subfamily vanilloid 1 and TRP subfamily ankyrin 1, respectively) (Xie 2009; Wei et al., 2010; Boillat et al., 2014; Horváth et al., 2015). Other TRP channels have also been found to be involved in CNI and pruritus such as TRPV3 (Lin et al., 2012) and TRPV4 (Zhao et al., 2014; Rajasekhar et al., 2015; Akiyama et al., 2016; Kim et al., 2016). In addition, the sensory nerves in the skin also express GPCRs, of which the protease-activated receptor (PAR) family in particular is known to be involved in CNI, especially PAR-2 and PAR-4 (Cocks and Moffatt 2000). Thus, TRPV1, TRPA1, PAR-2, and PAR-4 are associated not only with a rise in the intracellular Ca^2+^ concentration (iCa^2+^) with the subsequent exocytosis of neuropeptides but also with pro-inflammatory gene expression (Fig. 1). Moreover, following the activation of one of these receptors, intracellular pathways can lead to the sensitization of one of these channels.

In addition to their expression by sensory nerves, TRPV1, TRPA1, PAR-2, and PAR-4 are found in resident skin cells or cells recruited during CNI. Thus, they could contribute to the intense and narrow communication within the skin between sensory nerve endings, skin, and immune cells.

This review discusses the role of TRPV1 and TRPA1 activation or signaling pathways associated with their sensitization in the self-maintenance of CNI through the induction of a pro-inflammatory response in the skin.

## TRPV1

### A cationic channel with multiple direct roles in CNI induction and self-maintenance

TRPV1 is a nociceptive cationic (mainly Ca^2+^) channel responsive to high temperature (>43°C) (Boillat et al., 2014). In addition to excessive heat, various exogenous and endogenous triggering factors can directly activate or sensitize TRPV1 (Table 1). TRPV1 was initially described in cutaneous C- and Aδ-type sensory nerve endings, where it plays a critical role. It can be activated by the natural agonist capsaicin (Jancsó et al., 1967; Caterina et al., 1997). High temperature and capsaicin have been demonstrated to activate sensory nerves and induce neurogenic inflammation (Jancsó et al., 1967; Caterina et al., 1997). Indeed, TRPV1 activation by these direct activators allows the entry of Ca^2+^, leading to the release of neuropeptides, including SP (Andreev et al., 2012) and CGRP (Boillat et al., 2014), that control edema and can induce (or enhance) neurogenic inflammation (Szallasi and Blumberg 1989; Zygmunt et al., 1999; Steinhoff et al., 2003; Roosterman et al., 2006; Vincent et al., 2013).

**Table 1:** Endogenous and exogenous agonists involved in TRPV1 activation, sensitization, and inhibition

**Factors**	**Pathways of activation**	**References**
**Exogenous activators of TRPV1** ^**a**^:		
High temperature (>43°C) Protons (pH < 5.9)	**Direct activation:** -Inward current -iCa^2+^ elevation -Neuropeptide release -Inflammatory mediator release	(Boillat et al., 2014) (Caterina et al., 1997) (Tominaga et al., 1998)
Ultraviolet		(Lee et al., 2009, 2011)
Vanilloids: -Olvanil -Resiniferatoxin Natural substances: -Camphor -Piperin -Capsaicin		(Caterina et al., 1997) (Szallasi and Blumberg 1989)
**Endogenous activators of TRPV1:**		
Anandamide	**Direct activation:** -Inward current -iCa^2+^ elevation -Neuropeptide release -Inflammatory mediator release	(Smart et al., 2000) (Zygmunt et al., 1999)
AA^b^ metabolites: -NADA^c^ -ODA^d^ Lipooxygenase products: -HPETE^e^ -15-HPETE^f^ -LTB4^g^		(Huang et al., 2002) (Hwang et al., 2000)
DAG^h^ CAMKII^i^	Phosphorylation of TRPV1 (S502 and T704)	(Jung et al., 2004) (Woo et al., 2008)
PIP2^j^	Binding the extracellular leaflet (site: 777–820)	(Senning et al., 2014) (Ufret-Vincenty et al., 2015)
**Indirect activators/sensitizers of TRPV1:**		
Bradykinin Growth factors (e.g., NGF^k^ ) PgE2^l^	BK2R^p^ (GPCR^q^) RTK^r^ EPR^s^ (GPCR)	(Chuang et al., 2001) (Vellani et al., 2001) (Zhang et al., 2008)
Proteases	PAR-2 and PAR-4^t^ (GPCR)	(Amadesi et al., 2004, 2006) (Vellani et al., 2010)
Serotonin	5-HTR^u^ (GPCR)	(Sugiura et al., 2002)
Histamine	H1R and H4R^v^ (GPCR)	(Jian et al., 2016) (Shim et al., 2007)
ATP^m^	P2Y1R^w^ (GPCR)	(Numazaki et al., 2002) (Tominaga et al., 2001)
TRPA1^n^	Physical interaction or Ca^2+^ second messenger	(Anand et al., 2008) (Alpizar et al., 2013) (Spahn et al., 2014)
DAG	Indirectly by PKC^x^ activation	(Burgess et al., 1989) (Chuang et al., 2001) (Cesare et al., 1999)
**Inhibitors/desensitizers of TRPV1:**		
Calcineurin^o^	iCa^2+^-dependent dephosphorylation of TRPV1	(Docherty et al., 1996) (Jeske et al., 2006) (Patwardhan et al., 2006)
PIP2	Binding the intracellular leaflet (site: 777–820)	(Senning et al., 2014) (Ufret-Vincenty et al., 2015) (Woo et al., 2008) (Chuang et al., 2001)
TRPA1	Physical interaction or iCa^2+^ second messenger	(Akopian et al., 2007) (Patil et al., 2010)

Notes: ^a^ transient receptor potential vanilloid 1; ^b^ arachidonic acid; ^c^ N-arachidonoyl-dopamine ; ^d^ N-oleoyl dopamine; ^e^ 12-hydroperoxyeicosatetraenoic acid; ^f^ 15-hydroperoxy-eicosatetraenoic acid; ^g^ leukotrien B4; ^h^ diacylglycerol; ^i^ Ca^2+^/calmodulin-dependent kinase II; ^j^ phosphatidylinositol 4,5-bisphosphate; (iCa^2+^) intercellular Ca^2+^; ^k^ nerve growth factor; ^l^ prostaglandin E2; ^m^ adenosine triphosphate; ^n^ transient receptor potential ankyrin 1; ^o^ protein phosphatase 2B; ^p^ bradykinin B2 receptor; ^q^ G protein-coupled receptor; ^r^ receptor tyrosine kinase; ^s^ E prostanoid receptor; ^t^ protease-activated receptor-2 and 4; ^u^ 5-hydroxytryptamine receptor; ^v^ histamine receptors 1 and 4; ^w^ purinergic P2Y1 receptor; ^x^ protein kinase C

Furthermore, endogenous mediators produced or released during CNI (eicosanoids, acidosis, ATP, histamine, bradykinin, NGF) that further sensitize or activate TRPV1 on skin nerve terminals contribute to the self-maintenance of CNI (Roosterman et al., 2006). Moreover, TRPV1 was found to be expressed in skin cells (keratinocytes, dermal mast cells, dendritic cells, sebocytes, dermal blood vessels, hair follicles, and sweat glands), where it acts as a pain and chemical sensor (Ständer et al., 2004).

### TRPV1 sensitization/desensitization by endogenous modulators following intracellular pathway activation

#### Intracellular pathways of TRPV1 sensitization

The TRPV1 sensitization process is an intracellular mechanism that facilitates the gating of the channel or strengthens the currents evoked during the subsequent activation of TRPV1. The structure of TRPV1 was obtained by high-resolution cryo-electron microscopy (cryo-EM) and revealed multiple intracellular regulatory domains (Liao et al., 2013). TRPV1 is a polymodal receptor whose sensitization and endogenous regulatory pathways act via phosphorylation sites for kinases such as protein kinases C and A (PKC and PKA) and Ca^2+^/calmodulin dependent kinase II (CAMKII). PKC phosphorylates and sensitizes TRPV1 at the S502 and S800 phosphorylation sites (Numazaki et al., 2002; Bhave et al., 2003). The PKA phosphorylation sites S116, T370, S502, and T144 appear to sensitize TRPV1 and prevent its desensitization upon the repeated application of capsaicin (Bhave et al., 2002; Mohapatra and Nau 2003; Mohapatra and Nau 2005; Amadesi et al., 2006). Several studies have identified two specific phosphorylation sites for CAMKII on TRPV1 (S502 and T704) that modulate vanilloid binding. Thus, in human embryonic kidney-derived (HEK293) cells expressing Δ774–838-deleted CaMKII phosphorylation sites (Woo et al., 2008) or S502A/T704I mutants of TRPV1, capsaicin does not evoke a current, which suggests that phosphorylation by CaMKII is required to control the responsiveness of TRPV1 to the ligand (Jung et al., 2004).

Phospholipase C (PLC) activity goes through the decrease of phosphatidylinositol 4,5-bisphosphate (PIP2) level and through the production of diacylglycerol (DAG), both of which are involved in TRPV1 sensitization. Indeed, the correlation between PIP2 and the intracellular sensitization of TRPV1 has been established; both bradykinin and NGF (known to stimulate PLC pathways after binding to their cognate receptors) potentiated TRPV1 activity in a heterologous expression system and in dorsal root ganglion (DRG) neurons (Chuang et al., 2001). The role of PIP2 as a TRPV1 inhibitor or “desensitizer” after its depletion by sequestration or PLC hydrolysis has been subsequently shown to be essential for TRPV1 sensitization (Prescott and Julius 2003). In addition, the lack of a PIP2 binding site (786–828) increased the strong inward current evoked by a DAG analog and capsaicin in a Δ774–838 deletion mutant of TRPV1 (Woo et al., 2008). However, PIP2 could activate TRPV1 by binding the extracellular leaflet (777–820), while it has an inhibitor effect by binding the intracellular leaflet (682–725) (Senning et al., 2014; Ufret-Vincenty et al., 2015). Interestingly, in addition to its ability to directly activate TRPV1 via a PKC-independent pathway by binding to the capsaicin binding site at Y511; (Woo et al., 2008)), DAG could also contribute to the indirect sensitization of TRPV1 via PKC phosphorylation at the S502 and S801 sites (Burgess et al., 1989; Cesare et al., 1999; Chuang et al., 2001) (Numazaki et al., 2002; Bhave et al., 2003).

Finally, it might be noticed that the prolonged or repeated activation of TRPV1 induces a desensitization or inhibition process. It has been observed that the repeated activation of TRPV1 by chemical stimuli results in its desensitization by a Ca^2+^-dependent process (Chuang et al., 2001; Bhave et al., 2002; Dai et al., 2004). Protein phosphatase 2B (calcineurin) acts as a desensitizer of TRPV1, as indicated by the inhibition of calcineurin by cyclosporine or CsA-CyP, which have been shown to inhibit the desensitization of TRPV1 induced by capsaicin (Docherty et al., 1996; Mohapatra and Nau 2005). The cannabinoid WIN 55,212-2 seems to play an anti-inflammatory and analgesic role via the inhibition of TRPV1 by dephosphorylating the T144 and T370 sites in a Ca^2+^/calcineurin-dependent manner, reducing the release of CGRP (Patwardhan et al., 2006; Jeske et al., 2006). These data show the potential effects of calcineurin inhibitors on TRPV1 desensitization and suggest cannabinoids as new therapeutic drugs for CNI, hyperalgesia, itching, and pain.

#### Inflammatory mediators that use TRPV1 intracellular sensitization pathways

Following tissue damage, endogenously released inflammatory mediators (ATP, bradykinin, serotonin PGs, NGF, chemokines, histamine or proteases) can regulate TRPV1 activity via intracellular pathways associated with their specific GPCR. For example, in transfected HEK293 cells and DRG neurons, ATP increased capsaicin-induced TRPV1 activation via the purinergic receptor P2Y1, which potentiated TRPV1 activity in a PKC-dependent pathway (Tominaga et al., 2001; Numazaki et al., 2002). In the same way, in a Ca^2+^- and PLC/PKC-dependent manner, bradykinin, serotonin (G protein-coupled 5-HT receptor), PgE2 (G protein-coupled EP receptors), and NGF enhanced capsaicin-, heat-, proton-, and anandamine-evoked currents (Vellani et al., 2001; Sugiura et al., 2002; Zhang et al., 2008; Wilson et al., 2011). Similarly, the chemokine CCL3 was found to sensitize TRPV1 by increasing the heat-, anandamide-, and capsaicin-evoked Ca^2+^ influx through a PLC- and PKC-pathway in HEK293 cells and DRG neurons (Zhang et al., 2005).

Finally, histamine enhanced the capsaicin-mediated inward current, increased the Ca^2+^ level and then induced itching via sensory neurons, and this effect was abolished by TRPV1 antagonists and in TRPV1-deficient mice (Shim et al., 2007). These mechanisms appeared to involve phospholipase A2 (PLA2)-, lipoxygenase (NDGA)- and PLC-dependent pathways, with the subsequent sensitization and activation of TRPV1 (Jian et al., 2016). These studies suggest that TRPV1 is involved in histamine-dependent itching with arachidonic acid metabolites, primarily 12-hydroxyeicosatetraenoic (12-HETE), acting as a central participant (Shim et al., 2007).

Because of their ability to cleave and thus activate specific PARs, endogenous proteases play a specific role in CNI. Both PAR-2 and PAR-4 are known to induce CNI and therefore to be involved in several skin disorders, such as pruritus and AD (Asfaha et al., 2007; Briot et al., 2009; Vellani et al., 2010; Fu et al., 2014). In addition to their ability to induce CNI via the release of SP and CGRP, it has clearly been established that PAR-2 and PAR-4 activation could lead to TRPV1 sensitization. Patch clamp and Ca^2+^ imaging assays in HEK293 cells that co-express TRPV1 and PAR-2 and DRG neurons revealed that PAR-2 activation potentiates TRPV1 via phosphorylation by PKC- and PKA-dependent pathways (Amadesi et al., 2004; Dai et al., 2004; Amadesi et al., 2006). As a consequence, TRPV1 sensitization by a PAR-2 agonist reduced the temperature threshold for TRPV1 from 42°C to 33°C, leading to thermal hyperalgesia, pain, and inflammation (Dai et al., 2004; Amadesi et al., 2006; Amadesi et al., 2006). In addition, PAR-2 agonists potentiated the capsaicin-evoked CGRP and SP release from sensory neurons and thus enhanced CNI and hyperalgesia (Hoogerwerf et al., 2001; Amadesi et al., 2004). On the other hand, the involvement of PAR-4 activation in TRPV1 sensitization has been poorly studied. One study demonstrated that PAR-4 activation potentiated TRPV1 activation via a PKC-dependent pathway (Vellani et al., 2010; Patricio et al., 2015).

Altogether, mediators released in inflamed skin could contribute to peripheral sensitization, especially via TRPV1 sensitization, thus facilitating pain and itching. TRPV1 activation and/or sensitization can enhance peripheral inflammatory responses via the expression and release of other inflammatory mediators.

### Pro-inflammatory responses induced by TRPV1 activation

In addition to the clearly established role of TRPV1 in the release of neuropeptides from sensory neurons, its ability to increase the expression of inflammatory genes potentially involved in CNI, such as those for inflammatory cytokines, PGs and MMPs, has been shown in a few studies. The activation of TRPV1 by protons led to up-regulation of the expression of CGRP via CaMKII and CREB activation in DRG neurons. In addition, the proton-mediated induction of inflammatory pain and CGRP expression in DRG neurons was inhibited in TRPV1-deficient mice (Nakanishi et al., 2010). Another study showed that TRPV1 activation by capsaicin induced the PKC pathway-mediated up-regulation of TRPV1 and CGRP expression in acute CNI and increased PAR-4 mRNA and protein levels via a cAMP/PKA signaling pathway in cultured primary DRG neurons (Chen et al., 2013). Thus, it appears that a narrow interactive relationship between PAR-4 and TRPV1 exists in the development of NCI or in nociception from primary afferent neurons (Russell et al., 2010; Vellani et al., 2010; Chen et al., 2013; Gouin et al., 2015).

In addition to neuropeptide release, TRPV1-mediated Ca^2+^ influx in the skin could induce the expression or the release of other pro-inflammatory mediators and affect skin immune cells, contributing to the self-maintenance of CNI and/or cutaneous chronic inflammation.

In addition to TRPV1 activation in the sensory nerves, TRPV1 activation in human epidermal keratinocytes by capsaicin, acidification or ultraviolet (UV) radiation evoked an increase in the iCa^2+^ concentration (Inoue et al., 2002; Lee et al., 2009). In fact, several studies have shown that the exposure of keratinocytes to capsaicin or UV radiation leads to TRPV1-mediated Ca^2+^ influx, followed by the increased expression of cyclo-oxygenase (COX)-2, inflammatory mediators (e.g., interleukin (IL)-1β, IL-2, IL-4, and IL-8, tumor necrosis factor (TNF)-α, PGE2, and LTB4, as well as several MMPs, including MMP-13, 9, 3, and 2) (Inoue et al., 2002; Southall et al., 2003; Lee et al.. 2009; Jain et al., 2011; Lee et al., 2011). In an epithelial airway cell line, TRPV1 activation by capsaicin evoked the expression and release of thymic stromal lymphopoietin (TSLP) via Ca^2+^ elevation and calcineurin-mediated NFAT activity (Jia et al., 2014). For keratinocytes, no relation between TSLP secretion and TRPV1-mediated Ca^2+^ influx has been demonstrated, and this association needs to be investigated. Altogether, these mediators released by keratinocytes cause erythema, pain, and a UV-induced skin response and thickening (Lee et al., 2009; Jain et al., 2011; Lee et al., 2011). Moreover, they are known to sensitize TRPV1 in nociceptive neurons, with subsequent CGRP release and CNI (Hwang et al., 2000; Oprée and Kress 2000; Binshtok et al., 2008).

Immune cells present in the skin, such as mononuclear cells, dendritic cells (DCs) and mast cells, also express TRPV1, and TRPV1 activation directly affects the function of these cells (Saunders et al., 2009; Basu and Srivastava 2005; Ständer et al., 2004). Indeed, activation of TRPV1 in mononuclear cells induced cell death, and this response was reversed by a TRPV1 antagonist (Saunders et al., 2009). Capsaicin promotes DC maturation, which is abrogated in TRPV1^−/−^ DC cells. In an *in vivo* mouse model, injected capsaicin mediated the maturation and migration of skin DCs to the draining lymph nodes in TRPV1^+/+^ but not in TRPV1^−/−^ mice, confirming an important role for this channel in the innate immunity process (Basu and Srivastava 2005). Nevertheless, the potential inflammatory role of TRPV1 in DCs remains controversial, as evidenced by another study that did not find TRPV1 expression in DCs or the induction of calcium elevation by capsaicin. However, the neuropeptide SP activated DCs by eliciting robust Ca^2+^ elevation, suggesting that DC maturation and migration are dependent on CNI. Taken together, the potential inflammatory role of TRPV1 in DCs remains unclear and needs to be investigated. The involvement of mast cell degranulation during CNI in response to capsaicin is a related process that occurs indirectly via the release of peptide transmitters from sensory neurons (Bunker et al., 1991; Frydas et al., 2013). Nonetheless, TRPV1 activation by capsaicin has evoked Ca^2+^ elevation in numerous mast cell lines. Capsaicin-elicited Ca^2+^ did not induce mast cell degranulation but triggered the release of IL-4 (Bíró et al., 1998), which is known to be involved in AD associated with the recruitment of neutrophils, macrophages, CD3^+^ lymphocytes, and epidermal dendritic T lymphocytes (Zhao et al., 2016).

TRPV1 is also expressed in endothelial cells and smooth muscle cells, and its activation induces vasorelaxation by releasing nitric oxide (NO) by a Ca^2+^ influx-dependent mechanism (Yang et al., 2010; Ching et al., 2011; Himi et al., 2012). Endothelium-dependent vasodilatation is in turn dependent on endothelial nitric oxide synthase (eNOS) and TRPV1, as evidenced by the inhibition of capsaicin-evoked dilatation by an eNOS inhibitor (L-NAME) and a TRPV1 antagonist (capsazepine). These results confirmed that endothelial TRPV1 can induce vasodilatation via NO production (Kark et al., 2008; Yang et al., 2010). In addition to NO production, thermal endothelial-TRPV1 activation stimulated the endothelial cell-derived release of CGRP from endothelial cell lines, which contributed to the attenuation of endothelial cell damage (Ye et al., 2007; Luo et al., 2008). The CGRP receptor expressed on endothelial cells has been shown to have anti-inflammatory effects by inhibiting the production of the chemokines CXCL1, CCL2, and IL-8 (Huang et al., 2011). Taken together, these results indicate that endothelial TRPV1 acts as an anti-inflammatory factor both directly by inducing vasodilation via NO production and indirectly by inhibiting leukocyte recruitment via CGRP production.

In summary, TRPV1 activation mediates a narrow communication between sensory nerve endings, skin non-immune and immune cells, increasing release of inflammatory mediators (mainly cytokines and neuropeptides). These mediators act directly via specific receptors on neighboring cells, inducing the intracellular sensitization of TRPV1 and its activation. Hence, TRPV1 should be considered a central modulator of the self-maintenance of CNI.

## TRPA1

### Another cationic channel with multiple direct roles in neurogenic inflammatory dermatitis and pruritus

 TRPA1 is also a nociceptive cationic (mainly Ca^2+^) thermo-responsive channel that, in contrast to TRPV1, is implicated in cold thermal sensation (i.e., temperatures below 17°C). Besides cold stimuli, a variety of exogenous and endogenous activators or sensitizers of TRPA1 have been identified (Table 2).

**Table 2:** Endogenous and exogenous agonists involved in TRPA1 activation, sensitization, and inhibition

Factors	Pathways of activation	References
**Exogenous activators of TRPA1** ^**a**^:		
Cold temperature (<17°C)	**Direct activation:** -Inward current -iCa^2+^ elevation -Neuropeptides releases -Inflammatory mediators release	(Bíró and Kovács 2009)
Mustard oil compounds: -AITC^b^ -Benzyl isothiocyanate -Phenylethyl isothiocyanate -Isopropryl isothiocyanate -Methyl isothiocyanate Cinnamon compounds: -Cinnamaldehyde Garlic compounds: -Allicin Clove bud oil compounds: -Eugenol THC^c^		(Calixto et al., 2005) (Macpherson et al., 2005) (Chung et al., 2014)
Environmental pollutants: -Acrolein -Formalein		(Bautista et al., 2006) (McNamara et al., 2007)
TRPM8^d^ activators: -Icilin -Menthol		(Story et al., 2003)
Histamine-independent pruritogens: -Chloroquine -BAM8-22^e^ -AEW^f^		(Imamachi et al., 2009) (Mishra and Hoon 2010) (Mishra et al., 2011) (Wilson et al., 2011, 2013a, b)
**Endogenous activators of TRPA1:**		
ROS^g^ NO^h^ Lipid oxidation-derived: -PGA2^i^ -15dPGJ2^j^ -Δ12-PGJ2^k^	**Direct activation:** -Inward current -iCa^2+^ elevation -Neuropeptides releases -Inflammatory mediators release	(Pertovaara and Koivisto 2011) (Bautista et al., 2006)
Lipid oxidation products: -4-HNE^l^ -4-ONE^m^		(Taylor-Clark et al., 2009) (Engel et al., 2011)
DAG^n^		(Trevisani et al., 2007) (Bandell et al., 2004) (Jordt et al., 2004)
**Indirect activators/sensitizers of TRPA1:**		
Bradykinin	BK2R^t^ (GPCR^u^)	(Bandell et al., 2004) (Wang et al., 2008a)
Growth factors (e.g., NGF^o^ and BDNF^p^)	RTK^v^	(Malin et al., 2011)
Proteases	PAR-2 and PAR-4^w^ (GPCR)	(Dai et al., 2007) (Patricio et al., 2015)
Low iCa^2+^ concentration	EF-hand Ca^2+^-binding domain	(Sura et al., 2012) (Akopian et al., 2007)
Cytokines (e.g., TSLP^q^)	TSLP receptor (RTK)	(Wilson et al., 2013a, b)
TRPV1^r^	Physical interaction or Ca^2+^ second messenger	(Honda et al., 2014)
**Inhibitors/desensitizers of TRPA1:**		
High iCa^2+^ concentration	EF-hand Ca^2+^-binding domain	(Sura et al., 2012) (Wang et al., 2008b)
PIP2^s^	Direct binding to TRPA1	(Karashima et al., 2008) (Kim et al., 2008)
TRPV1	Physical interaction or Ca^2+^ second messenger	(Akopian et al., 2007) (Staruschenko et al., 2010) (Salas et al., 2009)

Notes: ^a^ transient receptor potential ankyrin 1; ^b^ allyl isothiocyanate; ^c^ tetrahydrocannabinol; ^d^ transient receptor potential melastatin 8; ^e^ bovine adrenal medulla 8-22; ^f^ acetone, ether and water; ^g^ reactive oxygen species; ^h^ nitric oxide; ^i^ prostaglandin A2; ^j^ 15-deoxy-delta12,14-prostaglandin J2; ^k^ Δ12-prostaglandin J2; ^l^ 4-hydroxynonenal; ^m^ 4-oxo-2-nonenal; ^n^ diacylglycerol; ^o^ nerve growth factor; ^p^ brain-derived neurotrophic factor; ^q^ thymic stromal lymphopoietin; ^r^ transient receptor potential vanilloide 1; ^s^ phosphatidylinositol 4,5-bisphosphate; ^t^ bradykinin B2 receptor; ^u^ G protein-coupled receptor; ^v^ receptor tyrosine kinase; ^w^ protease-activated receptor-2 and 4

The presumption that TRPA1 is involved in CNI is growing. Indeed, TRPA1 has been found to be co-expressed with TRPV1 and neuropeptides (SP and CGRP) in a subset of nociceptive sensory neurons (Vellani et al., 2010; Story et al., 2003). Low temperature has been demonstrated to activate sensory nerves and induce neurogenic inflammation (Story et al., 2003; Obata et al., 2005). Indeed, TRPA1 activation stimulated (or increased) neuropeptide (SP and CGRP) release from sensory neurons in a Ca^2+^-dependent manner, with subsequent signs of CNI, such as edema and leukocyte infiltration (Story et al., 2003; Meseguer et al., 2014; Trevisani et al., 2007; Thorne et al., 1991; Silva et al., 2011). In addition, a specific TRPA1 antagonist attenuated the capsaicin (a TRPV1 agonist)-evoked increase of cutaneous blood flow adjacent to an injury site in the plantar skin of rats, strongly suggesting that both TRP channels could cooperate in CNI induction (Wei et al., 2010).

TRPA1 is required for AD and histamine-independent chronic itching. Indeed, in acute oxazolone-induced dermatitis in mouse ears, TRPA1-deficient mice or WT mice treated with the TRPA1 antagonist HC-030031 showed diminished skin inflammation, which was associated with a decrease in ear epidermal thickness, eosin-positive cells, and CD4^+^ and CD8^+^ T cells in the ear tissue. TRPA1-deficient mice also diminished SP- and oxazolone-evoked scratching behavior and AD responses, as revealed by a decrease of dermatitis score, including erythema, scaring, excoriation, and swelling (Liu et al., 2013). Histamine-independent pruritus evoked by the TRPA1 agonists chloroquine, BAM8–22 or AEW was abrogated in neurons from TRPA1-deficient mice, impairing the scratching behavior response and increasing epidermal thickness (Wilson et al., 2011; Wilson et al., 2013a). In addition, the inflammatory cytokine TSLP is highly expressed in skin epithelial cells from AD patients and induces (or enhances) skin inflammation via T cells, DCs, and mast cell activation (Ziegler et al., 2013; Moniaga et al., 2013). Within the skin, TSLP also activates sensory neurons via TSLPR, and, subsequently, TRPA1 stimulation via a PLC-mediated pathway, thereby evoking robust itching in mice (Wilson et al., 2013b). To date, there is no evidence that TSLP and TRPA1 can induce CNI by releasing neuropeptides.

Similar to TRPV1, TRPA1 is also under the control of an intracellular sensitization process involving several inflammatory mediators, such as growth factors, bradykinins, proteases, and cytokines. By its localization in neuronal and non-neuronal skin cells, TRPA1 potentiates neurogenic skin inflammation by enhancing cellular responses (Inoue et al., 2002; Bautista et al., 2013).

### TRPA1 sensitization/desensitization by endogenous modulators following intracellular pathway activation

In a manner similar to that of TRPV1, TRPA1 activity is controlled by desensitization and sensitization processes. The repeated activation of TRPA1 by chemical stimuli results in the Ca^2+^- and current-dependent desensitization of TRPA1 (Story et al., 2003; Malin et al., 2011; Wilson and Bautista 2014). TRPA1 is a polymodal receptor with multiple intracellular ligand binding and phosphorylation sites that are involved in the activation and sensitization of this channel, respectively. Unlike most TRP channels, TRPA1 covalently interacts with many of its agonists through its N-terminal region, which contains cysteine and lysine residues involved in its direct activation (Hinman et al., 2006; Macpherson et al., 2007). Direct mechanisms of TRPA1 activation have been described, although the precise mechanisms of sensitization/desensitization by which the inflammatory mediators potentiate the channel activity remain poorly understood. Recent data, using cryo-EM, provided for the first time the structure of TRPA1 and shown several similarities with TRPV1 (Paulsen et al., 2015). It is appeared that the NH2-terminal of TRPA1 has approximately 15 ankyrin repeats with several cysteine and lysine residues crucial for activation by reactive agonists (Hinman et al., 2006; Macpherson et al., 2007). A EF-hand domain implicated in calcium-dependent gating was also found in its NH2 region (Zurborg et al., 2007; Doerner et al., 2007). Other specific domains have also been described such as a PIP2 binding site as well as CAMKII and PKC phosphorisation site (Choi et al., 2014). The transmembrane domain S6 are essential for gating by antagonists and agonists (Chen et al., 2008).

#### iCa^2+^ potentiates TRPA1 activation

Growing evidence suggests that Ca^2+^ regulates TRPA1 by potentializing and inactivating its activity at low and high intracellular concentrations, respectively (Jordt et al., 2004; Doerner et al., 2007). On the one hand, Ca^2+^ potentiated cinnamaldehyde-, AITC-, and carvacrol-evoked currents in HEK293 cells expressing TRPA1. In addition, an increase in iCa^2+^ also seems to directly act on the activation of TRPA1 by eliciting a current in a PLC-independent manner and serves as a co-agonist with icilin that directly interacts with TRPA1 (Zurborg et al., 2007; Doerner et al., 2007; Wang et al., 2008b). On the other hand, Ca^2+^ could also inactivate TRPA1. Indeed, even though high extracellular Ca^2+^ concentrations could enhance a cinnamaldehyde-evoked current, they could also induce rapid inactivation, which has not been found at low extracellular Ca^2+^ concentrations (Wang et al., 2008b). Hence, Ca^2+^ appears to be a crucial factor for the regulation of TRPA1 activity, suggesting that other TRP channels and GPCRs could modulate TRPA1 activity by mobilizing iCa^2+^.

#### Intracellular sensitizers of TRPA1

Several studies suggest that TRP channels may be potentiated by endogenous DAG and IP3, the products of PIP2 breakdown, by Ca^2+^ release from internal stores, by PKC activation or by the formation of endogenous lipid oxidation-derived and products in sensory neurons (Wang et al., 2008a; Mizumura et al., 2009). TRPA1-expressing CHO cells were also sensitive to DAG, arachidonic acid, and iCa^2+^ (Jordt et al., 2004; Bandell et al., 2004). PIP2 has an inhibitory effect not only on TRPV1 activity (refer to section Intracellular pathways of TRPV1 sensitization) but also on TRPA1 activity, since PIP2 has been shown to inhibit AITC- and mustard oil-evoked currents (Kim et al., 2008; Karashima et al., 2008). Moreover, the reduction of the PIP2 level at the membrane by polyphosphatase (e.g., PPPi), PIP2 antibody or phenylarsine oxide enhanced the AITC- and mustard oil-evoked currents via TRPA1 sensitization (Kim et al., 2008; Karashima et al., 2008). Taken together, these data suggest that TRPA1 activity could be sensitized by the activation of numerous GPCRs or receptor tyrosine kinases (RTKs) via the cAMP/PKA and PLC/PKC pathways following Ca^2+^ elevation (Taylor-Clark et al., 2008; Andersson et al., 2008; Taylor-Clark et al., 2009)

#### TRPA1 sensitization by inflammatory mediators

Several inflammatory mediators, such as growth factors, bradykinins, proteases, and, more recently, the inflammatory cytokine TSLP, have been found to act indirectly on TRPA1 activity and expression through specific receptor-dependent signaling pathways (Diogenes et al., 2007; Dai et al., 2007; Wang et al., 2008a; Malin et al., 2011). It has now been clearly established that TRPA1 in sensory neurons mediates the CNI responses to bradykinin (Jordt et al., 2004; Bandell et al., 2004; Bautista et al., 2006; Wang et al., 2008a), NGF, and brain-derived neurotrophic factor (BDNF) (Malin et al., 2011) by potentiating the activity of the channel and impairing its desensitization after repeated activation. In addition, NGF has been found to up-regulate the expression of TRPA1 mRNA via p38 MAPK activation in trigeminal ganglia and sensory neurons. Taken together, these mediators enhance the activity and prevent the desensitization of TRPA1 and consequently facilitate pain, hyperalgesia, and allodynia (Obata et al., 2005; Diogenes et al., 2007). TSLP released from keratinocytes potentiated TRPA1 activity by binding to its specific receptor (TSLPR) on sensory neurons in the skin of AD patients and in mouse models of AD. While TSLP-evoked sensory neuron activation and consequent chronic itching in AD has been established, the role of TSLP-triggered neuropeptide release in neurons and the subsequent CNI remain an open question (Wilson et al., 2013a, b).

While the involvement of endogenous proteases in TRPV1 sensitization via PAR-2 and PAR-4 activation is well established (refer to section Inflammatory mediators that use TRPV1 intracellular sensitization pathways), the implication of these proteases in TRPA1 sensitization remains poorly understood. PAR2 activation has been demonstrated to enhance TRPA1 agonist-evoked pain behavior in rats via PLC activation and subsequent PIP_2_ hydrolysis (Dai et al., 2007). In addition, PAR-2-evoked mechanical and cold allodynia, as well as heat hyperalgesia, was found to be dependent on the PKA and PKC signaling pathways, which sensitize TRPA1 (Chen et al., 2011). The involvement of PAR-4 in the sensitization of TRPA1 is poorly studied, but a recent finding demonstrated its potential role in pruritus: the dorsal intradermal administration of the PAR-4 agonist peptide AYPGKF-NH_2_ elicited intense scratching behavior via a SP release in mice, which was abolished by the TRPV1 and TRPA1 antagonists SB366791 and HC-030031, respectively (Patricio et al., 2015), but not in TRPA1-deficient mice. An opposite pattern in PAR-4-evoked itching via TRPA1 by compensatory mechanisms in TRPA1^−^ deficient mice has been hypothesized (Patricio et al., 2015; Petrus et al., 2007). Consequently, PAR-4 activation could sensitize TRPA1 in collaboration with TRPV1 and elicit itching behavior via the release of neuropeptides from sensory neurons; however, some points still require clarification, mainly the intracellular pathways underlying the TRPV1/TRPA1 sensitization via PAR-4 activation (Patricio et al., 2015).

Finally, the intracellular pathways for many inflammatory mediators could sensitize TRPA1 in injured skin, contributing to channel activity and impairing the TRPA1 desensitization, thereby enhancing peripheral inflammatory responses via the production of other inflammatory mediators.

### A pro-inflammatory response induced by TRPA1 activation

In addition to its ability to induce the release of neuropeptides from sensory neurons, TRPA1 seems to play an important role in the modulation of numerous genes that could amplify the cutaneous inflammatory process. The involvement of TRPA1 in the regulation of 1,843 (2,423 probe sets) genes in whole trigeminal ganglia isolated from AEW-treated mice has been established (Wilson et al., 2013a). In this model, TRPA1 activation by AEW highly up- or down-regulated the expression of several cytokines, plasmatic receptors, and ion channels and affected growth regulation, intracellular pathways, and immune cell specificity proteins. For example, AEW up-regulated the inflammatory bradykinin receptor (BK2R) by 2-fold and up-regulated several itch receptors such as MRGPR and PAR-2 in the trigeminal ganglia neurons (Wilson et al., 2013a). These three receptors sensitize and open TRPA1. Thus, the up-regulation of itch and inflammatory receptors in sensory neurons strongly indicates a role for TRPA1 in chronic pruritus and CNI via the enhancement of sensory neuron sensitivity to itch and inflammatory mediators.

Growing evidence indicates that TRPA1 acts as an inflammatory regulator. The results of recent studies correlated the activation of TRPA1 with expressional changes in the skin, as associated with cutaneous inflammation, AD, and pain that is blocked in TRPA1-deficient mice. Indeed, TRPA1 activation by oxazolone induced chronic dermatitis in mouse ears and concomitant up-regulation of inflammatory cytokines (i.e., IL-1β, IL-4, and IL-16 and chemokine (C-X-C motif) ligand 2 (CXCL-2)), neuropeptides (i.e., SP and endothelin (ET-1)), nerve growth factor (NGF), and neurotransmitters (i.e., serotonin) known to induce AD, pruritus, and pain and increase nerve fiber density (Liu et al., 2013). Additional studies established the expressional change of 9,340 genes linked to itching associated with TRPA1 in skin biopsies from AEW-treated mice, showing that TRPA1 up-regulated the expression of 79% of itch-related genes known to be involved in the initiation and maintenance of chronic itching as well as pruriginous skin disorders (e.g., the IL-31 receptor (IL-31RA), aquaporin 3, and IL-33 (Wilson et al., 2013a; Nakahigashi et al., 2011; Nobbe et al., 2012; Olsson et al., 2006; Sonkoly et al., 2006). In these studies, the expressional changes induced by oxazolone or AEW were abrogated in the TRPA1^−/−^ mice and in WT mice treated with TRPA1 antagonists (Wilson et al., 2013a; Liu et al., 2013). These data strongly suggest the involvement of TRPA1 in pruritus.

TRPA1 is also expressed in the basal keratinocytes, where it acts as a pain, thermal, and chemical sensor (Atoyan et al., 2009). TRPA1 was found to modulate inflammatory gene expression in keratinocytes by increasing the expression of IL-1α and IL-1β (Atoyan et al., 2009) and cause the secretion of PgE2 (Jain et al., 2011). Both IL-1 and PgE2 are known to be involved in skin inflammation and itching by decreasing the mechanical threshold and the thermal responsiveness of the sensory nerve endings, which could facilitate CNI (Binshtok et al., 2008). In addition, in keratinocytes, TRPA1 activation increases heat shock protein (HSP) 27, which is known to up-regulate the expression of inflammatory cytokines such as IL-1β, TNF-α, and IL-6 in the skin during murine allergic contact hypersensitivity (Yusuf et al., 2009; Atoyan et al., 2009). Altogether, these findings indicate that TRPA1 activation contributes to the production of several cytokines from keratinocytes that directly trigger or enhance CNI by acting on neighboring target cells.

In addition to keratinocytes, TRPA1 acts on skin immune cells, but it appears to have an anti-inflammatory role in monocytes/macrophages. The TRPA1 activators cinnamaldehyde and carvacol have been found to inhibit bacterial lipopolysaccharides (LPS)-induced nuclear factor-kappa B (NF-κB)-mediated promoter activity and reduce the expression of iNOS, COX2, and TNF-α and subsequent NO production in macrophages (Chao et al., 2008; Romano et al., 2013). Similarly, cinnamaldehyde inhibited bacterial LPS- and lipoteichoic acid (LTA)-mediated IL-1β, IL-6, and TNF-α release in murine macrophages and human blood monocytes, presumably through ROS production (Hsu and Wen 2002; Chao et al., 2008; Romano et al., 2013). Furthermore, an increase in TNF-α as well as the induction of both kinnitric oxide synthases (iNOS) and COX2, which are involved in the formation of NO and PGE2, respectively, are known to act in the pathogenesis of several inflammatory skin diseases, such as AD, psoriasis, and pruritus (Ormerod et al., 1998; Kim et al., 2015; Ostadhadi et al., 2015; Ahn et al., 2016; Sereflican et al., 2016). It would therefore appear that TRPA1 acts as an anti-inflammatory actor in monocytes and macrophages, but these data are controversial (Billeter et al., 2015). The activation of TRPA1 by cinnamaldehyde acts on adaptive immunity by suppressing lymphoproliferation and promoting T-cell maturation, as revealed by T-cell differentiation from CD4 and CD8 double-positive cells to CD4 or CD8 single-positive cells in LPS-treated mouse splenocytes (Koh et al., 1998). These results imply that TRPA1 could enhance inflammation and immunoreactivity by directly acting on lymphocyte differentiation.

TRPA1 activation by AITC has been found to trigger vasodilatation in rat cerebral arteries in an endothelium-dependent mechanism. TRPA1-mediated vasodilation triggers Ca^2+^ influx in rat endothelial arteries through Ca^2+^-activated potassium channels in endothelial cells and inwardly rectifying potassium channels in arterial myocytes (Earley et al., 2009; Qian et al., 2013). This mechanism suggests that this channel could also act in edema and vasodilatation during CNI, but this hypothesis must be verified. However, another study established that TRPA1 activation by cinnamaldehyde played an anti-inflammatory role by suppressing the attachment of leukocytes to endothelial cells (Liao et al., 2008).

To conclude, TRPA1 activation and sensitization mediate skin inflammation by increasing the release of inflammatory mediators, although their role in immune cells remains to be clarified. In turn, secreted factors mediate the intracellular sensitization of TRPA1, thus facilitating its activation, which in turn contributes to enhancement of the self-maintenance of CNI.

## CROSS-REGULATION BETWEEN TRPV1 AND TRPA1

In addition to co-expression in a subset of sensory nerves and non-neuronal cells (keratinocytes), a functional interaction between TRPA1 and TRPV1 (cross-sensitization/desensitization) has been established, which suggests the cooperation between these channels to promote inflammatory thermal hyperalgesia, CNI, and pain (Bautista et al., 2006; Akopian et al., 2007; Anand et al., 2008; Patil et al., 2010; Aubdool and Brain 2011; Spahn et al., 2014; Fischer et al., 2014).

This cross-communication between TRPA1 and TRPV1 appears to involve the concentration of cytosolic Ca^2+^, one of the main sensitizers/desensitizers of TRPA1 and TRPV1 (Jordt et al., 2004; Jung et al., 2004; Doerner et al., 2007; Woo et al., 2008). It has been shown that capsaicin- and mustard oil-evoked currents desensitized each other via iCa^2+^ elevation in TRPV1- and/or TRPA1-expressing CHO cells (Akopian et al., 2007). Another relevant study confirmed the role of the influx of Ca^2+^ via TRPV1 as a second messenger for the desensitization of TRPA1. Indeed, TRPA1 evoked a current in CHO cells expressing TRPA1, and this current was inhibited by the co-expression of TRPV1 (Patil et al., 2010). In addition, TRPA1 and TRPV1 were found to interact by forming a physical and functional interaction at the plasma membrane, as revealed by co-IP and FRET assays in TRPA1- and TRPV1-co-expressing CHO cells, as well as in sensory neurons (Akopian et al., 2007; Salas et al., 2009; Staruschenko et al., 2010; Fischer et al., 2014). In these cells, TRPV1 regulated the desensitization of TRPA1, which appeared to be independent of iCa^2+^ (Staruschenko et al., 2010).

On the other hand, evidence has established that the TRPV1 and TRPA1 channels also sensitize each other in a Ca^2+^-dependent signaling pathway. Indeed, bradykinin and facial capsaicin injections sensitized TRPA1 via TRPV1 in a Ca^2+^-dependent manner in cultured trigeminal neurons from mice and rats, leading to inflammatory cold hyperalgesia (Bautista et al., 2006; Honda et al., 2014). The opposite pattern was also found: TRPA1 could sensitize TRPV1 to AITC-, cinnamaldehyde-, and mustard oil-enhanced heat- and capsaicin-evoked currents and Ca^2+^ elevation in a Ca^2+^-, cAMP/PKA-, and TRPV1 phosphorylation-dependent manner in sensory neurons and in HEK293 cells co-expressing TRPV1 and TRPA1 (Anand et al., 2008; Alpizar et al., 2013; Spahn et al., 2014).

Altogether, the TRPV1-TRPA1 interaction and cross-communication via the iCa^2+^ concentration could inhibit or enhance the ability to a large variety of exogenous and endogenous activators to cause Ca^2+^ elevation, neuropeptide release and, consequently, CNI.

## CONCLUSIONS AND FUTURE DIRECTIONS

The TRPV1 and TRPA1 channels are at the core of the inflammatory process that occurs in various cutaneous neurogenic disorders that are pruritic diseases, such as psoriasis, AD, and Netherton syndrome. It is now clear that both TRP channels act far beyond the sensory nerves by potentiating the pathology of numerous skin disorders in non-neuronal skin cells, such as keratinocytes, mast cells, dendritic cells, and blood vessels. We still have a great deal to learn about these channels, notably about the change of gene expression in sensory neurons and non-neuronal skin cells, especially in relation to their role in the induction and self-maintenance of CNI (*in vitro* and *in vivo).* A great deal of knowledge about their interaction, their cross-sensitization, and their sensitization by inflammatory mediators awaits discovery, especially in relation to the potentiation and the chronicitization of skin disorders associated with CNI. Although the molecular mechanisms of TRPV1 and TRPA1 sensitization induced during CNI and itching are still poorly understood, their clinical relevance in pruritus, inflammatory thermal hyperalgesia, and pain are indisputable.

Targeting one or more of the intracellular signaling pathways of TRP channel sensitization may allow new opportunities for the treatment of skin disorders associated with CNI. Further studies are necessary to test the potential effects of treatment with protein signaling inhibitors in neurogenic inflamed skin. A new and interesting approach could also be to focus on the effect of the iCa^2+^ concentration to mediate TRP desensitization and thus block the self-maintenance of CNI.
